# Differential Effects of Wheat Bran Antioxidants on the Growth Dynamics of Human Cancer Cells

**DOI:** 10.3390/foods14091633

**Published:** 2025-05-06

**Authors:** Md Sharifur Rahman, Guangyan Qi, Cheng Li, Yonghui Li, Weiqun Wang, Anthony Atala, Xiuzhi Susan Sun

**Affiliations:** 1 Department of Biological and Agricultural Engineering, Kansas State University, Manhattan, KS 66506, USA; mdsharifur@ksu.edu; 2 Wake Forest Institute for Regenerative Medicine, Wake Forest University School of Medicine, Winston-Salem, NC 27101, USA; gqi@wakehealth.edu (G.Q.); aatala@wakehealth.edu (A.A.); 3 Department of Grain Science and Industry, Kansas State University, Manhattan, KS 66506, USA; chengli@ksu.edu (C.L.); yonghui@ksu.edu (Y.L.); 4 Department of Food, Nutrition, Dietetics and Health, Kansas State University, Manhattan, KS 66506, USA; wwang@ksu.edu

**Keywords:** wheat bran antioxidants, ferulic acid, hydrolyzed arabinoxylan oligomers, cancer cell growth, SW480, HepG2, HeLa, 3D culture, targeted cancer therapy

## Abstract

Wheat bran, rich in phenolic compounds like ferulic acid, possesses notable antioxidant properties that may contribute to cancer treatment strategies. This study examined the effects of hydrolyzed arabinoxylan oligomers (HAOs) linked with ferulic acid from hard wheat bran on three human cancer cell lines: colon cancer (SW480), liver cancer (HepG2), and cervical cancer (HeLa). Cells were cultured in a three-dimensional (3D) 0.5% PGS matrix and exposed to varying concentrations (100, 500, and 1000 μg/mL) of wheat bran antioxidants (WBA) extracts. Results show that WBA inhibited growth of SW480 cells, significantly reducing spheroid expansion and promoting dehydration. In contrast, HepG2 cells exhibited increased growth under WBA treatment, suggesting a non-toxic, growth-enhancing effect. No significant changes were observed in HeLa cell growth, with cell viability remaining high across all treatments. These findings highlight the selective influence of WBA on cancer cell behavior, underscoring its potential for targeted, personalized cancer therapies. This study provides valuable insights into the application of antioxidant-rich compounds for modulating specific cancer cell dynamics, paving the way for novel therapeutic approaches.

## 1. Introduction

Natural antioxidants, particularly phenolics and bioactive compounds derived from dietary sources, have gained considerable attention for their anti-inflammatory, anti-cancer, and anti-aging properties [1]. Phenolic-rich byproducts such as rice bran, grape pomace, and apple peel have been stabilized and utilized as low-cost materials to extract various valuable compounds for nutraceuticals, dietary supplements, and functional food formulations [2]. Wheat-based materials represent a cost-effective and abundant source of antioxidants [3]. Among wheat components, wheat bran, a byproduct of the flour milling process, can be utilized as a functional food and dietary intervention and contains the highest concentration of phenolic compounds, known for their potent in vitro antioxidant activity and their ability to prevent low-density lipoprotein oxidation and DNA damage [3,4]. Arabinoxylan (AX), a primary constituent of wheat bran, provides several health benefits, including antioxidant effects, modulation of gut microbiota, production of short-chain fatty acids, and anti-cancer properties [3,5]. AX’s antioxidant potential primarily stems from its attached phenolic acids, which stabilize free radicals through resonance [6,7]. However, the polymeric structure of AX can obscure these phenolic acids, reducing its antioxidant efficacy. Hydrolyzing AX into xylo-oligosaccharides (XOSs) via xylanase is thought to enhance antioxidant capacity compared to intact wheat bran [3]. Previous studies evaluating whole wheat bran may not accurately reflect the antioxidant potency of XOSs, as other components such as fat, starch, and protein can confound the results [3].

Antioxidants play a vital role in neutralizing reactive oxygen species (ROS)—highly reactive molecules that induce oxidative stress and damage essential cellular components, including DNA, lipids, and proteins [1,5,8]. The role of oxidative stress in cancer progression is well established, as excessive ROS can lead to genetic mutations, uncontrolled cell proliferation, and metastasis [9,10,11]. This has led to growing interest in the therapeutic potential of wheat bran antioxidants (WBA) to modulate cancer cell growth. Although numerous studies have documented the antiproliferative effects of dietary antioxidants on various cancer cell lines, the specific influence of wheat bran XOS on 3D-cultured cancer cells remains largely unexplored [1,12]. This study aimed to address that gap by investigating the effects of WBA on three cancer cell lines—SW480, HepG2, and HeLa.

Colorectal cancer, one of the most common cancer types globally, is represented by the SW480 cell line, derived from a primary adenocarcinoma of the colon and commonly used to model colorectal cancer progression in vitro [13,14]. Studies show that dietary antioxidants can inhibit colorectal cancer cell growth by modulating signaling pathways such as Wnt/β-catenin and PI3K/AKT, which are crucial for cell proliferation and survival [13,14]. For instance, antioxidants like quercetin and resveratrol induce apoptosis and inhibit colon cancer cell proliferation via ROS-mediated mechanisms [15]. Given WBA’s high antioxidant capacity, we hypothesize that it may exert similar growth-suppressive effects on SW480 cells by modulating ROS levels and related pathways. Additionally, liver cancer, represented by the HepG2 cell line, is the third leading cause of cancer-related deaths, particularly due to its aggressive nature and resistance to conventional treatments [16]. Previous research highlights the role of antioxidants in promoting liver cell survival while reducing tumor growth, suggesting that WBA may support healthy liver cell function while inhibiting malignant transformation [17,18]. For cervical cancer, HeLa cells are a widely recognized model, with studies indicating that antioxidants may disrupt cancer progression by targeting ROS-driven cellular mechanisms [19,20]. Although antioxidants like vitamin C and polyphenols have been examined in cervical cancer studies, their effects in a 3D culture environment—which closely mimics the tumor microenvironment—remains underexplored [20,21].

This study incorporates a 3D-embedded culture system using a 0.5% PGmatrix-Spheroids (PGS) matrix to provide a physiologically relevant assessment of how WBA affects cancer cell growth and spheroid formation in SW480, HepG2, and HeLa cell lines. Since 3D cultures better simulate the in vivo environment, this approach enables more accurate observations of cell–cell and cell–matrix interactions, which are essential for understanding cancer biology [22,23]. Furthermore, 3D systems establish gradients of oxygen, nutrients, and metabolites, which are critical for exploring mechanisms such as hypoxia, metabolic adaptation, and chemoresistance [24]. Through a comprehensive evaluation of WBA extract’s cytotoxic effects on SW480, HepG2, and HeLa cancer cell lines, this research aims to offer new insights into the role of antioxidants in cancer therapy and deepen our understanding of how antioxidants impact cancer cell biology.

## 2. Materials and Methods

### 2.1. WBA Extraction

Xylo-oligosaccharides linked with ferulic acid were extracted from hard red winter wheat bran (protein content: 16.8%), sourced from the Hal Ross Flour Mill in Manhattan, Kansas, following the method outlined by Li et al. (2024) [3]. The extraction process began with defatting the bran, where 4 mL of hexane per gram of bran was used for a 1 h extraction. The defatted bran was then air-dried in a fume hood. Subsequently, destarching was carried out using α-amylase (500 U/mg) at pH 6, followed by deproteinization with Alcalase (0.8 U/g) at pH 7.5 for 30 min at an optimal temperature of 60 °C. After deproteinization, the resulting protein hydrolysate was heat-inactivated by boiling. Xylanase was added to the pretreated bran in 20 mM sodium acetate buffer (pH 5.5), and the mixture was incubated at 50 °C for 12 h. Following incubation, the mixture was centrifuged at 8000× *g* for 15 min to separate the supernatant, which was then dialyzed at 4 °C for three days before being lyophilized to yield the xylo-oligosaccharide (HAO).

### 2.2. Cell Cultures

#### 2.2.1. Cell Lines

Human colorectal carcinoma SW480, human hepatocellular carcinoma HepG2 (HB-8065), and human cervical carcinoma HeLa cell lines were purchased from American Type Culture Collection (ATCC) (Manassas, VA, USA).

#### 2.2.2. Cell Culture Condition

The PGmatrix Spheroids (PGS) kit (PepGel LLC, Winston Salem, NC, USA) was used according to the manufacturer’s user guide. Briefly, cell suspension at a desirable seeding density in a selected medium was initially mixed with PGworks (PepGel LLC, Winston Salem, NC, USA). 1% PGS solution was then added to this mixture (the final concentration of PGS was 0.5%) and thoroughly blended. The resulting cell mixture was dispensed into a 24-well plate at 500 μL per well and incubated at 37 °C for 30 min to form a hydrogel. According to our preliminary testing, 0.5% PGS showed desirable gel strength to hold the cell spheroids suspended (no precipitation). Following gelation, the target medium was gently added to feed the cells. Then cells were fed as needed in the following days, as described in the cell line culture protocols in Section 2.4.

### 2.3. Cell Spheroids Harvesting and Dissociation

Cancer spheroids were harvested from the 3D-embedded culture following the manufacturer’s guidelines (PepGel LLC, Winston Salem, NC, USA). Briefly, to disrupt the gel, the gel and medium were pipetted 6–8 times, then transferred to a conical tube. To dilute the solution for separation, 20 volumes of DPBS without calcium were added. The mixture was centrifuged at 500× *g* at 24 °C for 5 min. After centrifugation, the supernatant was discarded, and cancer spheroid pellets were collected from the bottom of the tube. These spheroids were used for fixation and staining analysis.

For cell passage or cryopreservation, these spheroids were dissociated into single cells by incubating with 1 mL/well of trypsin EDTA 0.25% (5×) (ThermoFisher Scientific, Waltham, MA, USA, Catalog: 15400054) at 37 °C. Once sufficient cell dissociation was observed under a microscope, the trypsinization was stopped by adding culture medium and the dissociation time was recorded. Cells were washed and centrifuged for passage or cryopreservation as well as analysis or applications as needed.

### 2.4. Growth Performance of Cancer Cells with WBA Induction

#### 2.4.1. SW480 3D Culture, WBA Induction, and Harvesting

SW480 cells were cultured under 3D-embedded conditions in 0.5% PGS using DMEM complete medium (DMEM + 10% FBS + 1% P/S), as described in Section 2.2. In 1 well of a 24-well plate, a solution containing 2 × 10^4^ single cells in 240 µL of DMEM complete medium, 10 µL of PGworks, and 250 µL of 1% PGS was added. On day 3, 1 mL of old medium was carefully aspirated without disturbing the cells and 1.5 mL of fresh medium was added along with the required volume of WBA extract, as specified in Table 1. Cells were harvested on day 7 according to the procedure described in Section 2.3.

#### 2.4.2. HepG2 Culture, WBA Induction, and Harvesting

HepG2 cells were cultured under 3D-embedded conditions in 0.5% PGS using DMEM complete medium following the procedure described in Section 2.2. In 1 well of a 24-well plate, a solution containing 4 × 10^4^ single cells in 240 µL of DMEM complete medium, 10 µL of PGworks, and 250 µL of 1% PGS was added. On day 3, 1 mL of old medium was carefully removed without disturbing the cells, and 1.5 mL of fresh medium was added along with the required volume of WBA extract, as specified in Table 1. Cells were harvested on day 7 following the procedure in Section 2.3.

#### 2.4.3. HeLa Culture, WBA Induction, and Harvesting

HeLa cells were cultured in the 3D-embedded condition in 0.5% PGS using DMEM complete medium following the procedure described in Section 2.2. In 1 well of a 24-well plate, a solution containing 4 × 10^4^ single cells in 240 µL of DMEM complete medium, 10 µL of PGworks, and 250 µL of 1% PGS was added. On day 4, 1 mL of old medium was carefully removed without disturbing the cells, and 1.5 mL of fresh medium was added along with the required volume of WBA extract, as specified in Table 1. Cells were harvested on day 8 following the procedure in Section 2.3.

### 2.5. Cell Morphology and Image Analysis

Bright-field and fluorescence imaging were conducted using an Axio Vert A1 inverted microscope (Carl Zeiss Microscopy, Munich, Germany). Image processing and analysis were performed using FIJI-ImageJ 1.54p software [27].

### 2.6. Cell Count and Viability Measurement

Following the cell retrieval procedures outlined Section 2.3, cell count, and viability were assessed using the Auto2000 Cellometer (Nexcelom Bioscience LLC, Lawrence, MA, USA) with an acridine orange/propidium iodide (AO/PI) assay from Nexcelom Bioscience. A 20 μL aliquot of well-suspended cell solution was mixed with 20 μL of AO/PI reagent. The Cellometer, programmed for bright-field and dual-fluorescence imaging, detected live and dead cells: live cells fluoresced green, while apoptotic cells with early membrane damage fluoresced orange. The Cellometer calculated and reported cell count, diameter, viability, and concentration. Fold change was determined by dividing the total number of harvested cells by the initial seeding number, and viability was calculated by dividing the total number of live cells by the total number of harvested cells.

### 2.7. Hematoxylin and Eosin (H&E) Staining

Cancer cell spheroids were fixed overnight in 10% neutral buffered formalin, washed with DPBS supplemented with Ca^2+^/Mg^2+^ (Sigma-Aldrich, St. Louis, MO, USA, Catalog: D8662-1L), and subsequently embedded in 1% agar (Sigma-Aldrich, Catalog: A1296-100G) solution cooled to approximately 60 °C. The agar-embedded samples were then processed and embedded in a paraffin. These embedded samples were sectioned, mounted onto glass slides, dewaxed, and subjected to staining with hematoxylin and eosin, as well as imaging. Image processing was performed using FIJI ImageJ version 1.54p [27].

### 2.8. Statistical Analysis

All statistical analyses were conducted using Microsoft Excel and Minitab 20.3, with results presented as mean ± standard deviation (SD). Significance was evaluated using a one-way ANOVA with Tukey HSD multiple comparisons. Statistical significance was set at *p* ≤ 0.05. All experiments were performed in triplicate (*n* = 3) to ensure reproducibility of the results.

## 3. Results

### 3.1. WBA Alleviates SW480′s Growth Performance

**Figure 1:**
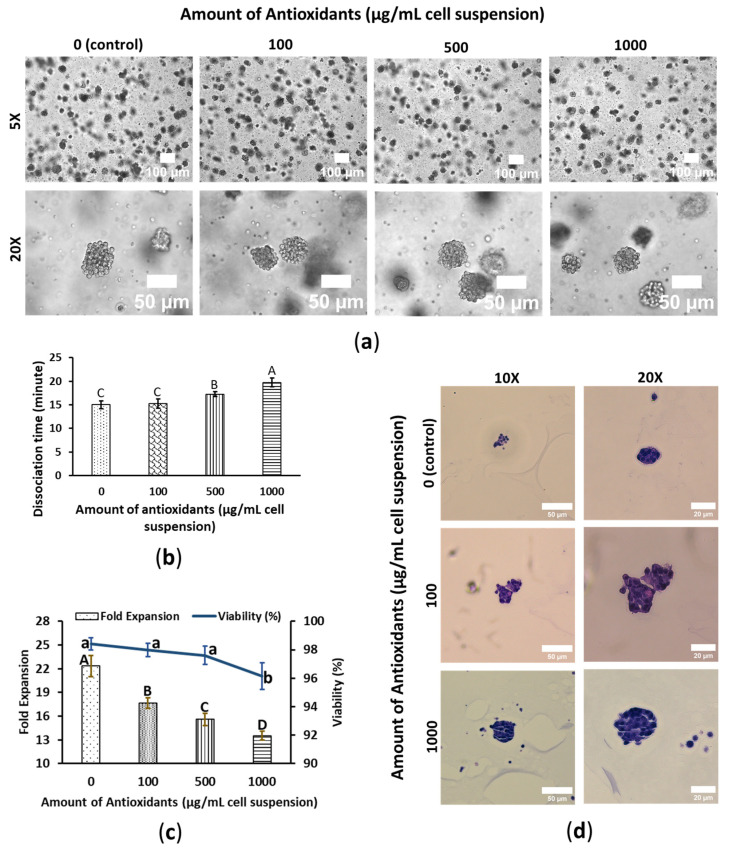
WBA mitigates SW480 colon cancer cell’s growth performance. Cells were cultured in 3D-embedded conditions using PGS (0.5%) in 24-well plates. Antioxidants were induced on day 3 and the cell was harvested on day 7. The cell spheroids shown in the figure are also on day 7. (**a**) Cell morphology of SW480. The scale bars are 100 and 50 μm; the resolutions are 5× and 20×. (**b**) Cell dissociation time with trypsin EDTA 0.25% at 37 °C. Data are shown as means ± SD of three independent biological replicates (*n* = 3). *p* < 0.05. The means that do not share a letter are significantly different. (**c**) Fold expansion and viability of SW480. The column chart shows fold expansion, and the line chart shows viability. Data are shown as means ± SD of three independent biological replicates (*n* = 3). *p* < 0.05. The means that do not share a letter are significantly different. The uppercase letters (A–D) represent the significant difference in fold expansion and the lowercase letters (a–b) represent the significant difference in viability. (**d**) Cell spheroid images of H&E stained WS480. The scale bars are 50 and 20 μm; the resolutions are 10× and 20×.

SW480 cells formed spheroids in a 3D PGS system, resembling a cluster of grapes, with well-hydrated, circularly shaped cells loosely arranged without forming any distinct hollow structures or specific cellular organization within the spheroids. In contrast, WBA treatment significantly altered the morphology of the SW480 cells. WBA-treated spheroids appeared more like clusters of dehydrated, squished grape raisins. The cells exhibited a more compressed and parched appearance, with the spheroids adopting a more compact circular shape. The degree of dehydration increased with concentrations of WBA. Consequently, the spheroid’s diameter decreased with WBA concentration: control spheroids had a diameter of approximately 40–60 μm, while 1000 μg/mL WBA-treated spheroids measured about 30 to 50 μm in diameter. Despite these morphological changes, no precipitation was observed in any treatment conditions. However, a noticeable reduction in spheroid count was observed at higher WBA concentrations (Figure 1a). On day 7, cells were harvested and treated with 0.25% trypsin-EDTA to dissociate into single cells, and dissociation times were recorded to evaluate WBA’s impact on cell detachment. Dissociation times increased significantly with higher WBA concentrations, with control and 1000 μg/mL WBA-treated SW480 cells showing times of 15 ± 0.82 and 19.75 ± 0.96 min, respectively. However, no effect on dissociation time was observed at lower concentrations, such as 100 μg/mL (Figure 1b).

The effects of WBA on cell fold expansion and viability were assessed after a 4-day exposure period. Cell viability remained relatively stable up to 500 μg/mL WBA but significantly decreased at 1000 μg/mL, although it still exceeded 95%, indicating a strong survival rate. In terms of cell growth, WBA-treated cells exhibited markedly reduced fold expansion compared to the control group, with a clear reduction corresponding to each increase in WBA concentration. The growth rate for control cells was 22.33 ± 1.31, while cells treated with 1000 μg WBA showed approximately 40% less growth, demonstrating that WBA exerts a toxic effect on SW480 colon cancer cells (Figure 1c). These findings also suggest that WBA possesses growth-suppressing effects in SW480 cells, highlighting its potential as a candidate for colon cancer treatment.

SW480 cells were subjected to H&E staining to assess cell pattern, shape, and structure within the spheroids. Due to significant cell dissociation during sample preparation, fewer spheroids were available for imaging, especially for control. Structural differences were observed between the control and WBA-treated SW480 cells. Control SW480 spheroids exhibited hyperchromatic nuclei, and a potentially higher nuclear-to-cytoplasmic ratio compared to WBA-treated cells. Nucleus size was decreased with an increase in WBA concentration, along with a reduction in individual cell diameter, resulting in greater intercellular distances within the WBA-treated spheroids (Figure 1d).

### 3.2. WBA Accelerates HepG2′s Growth Performance

**Figure 2:**
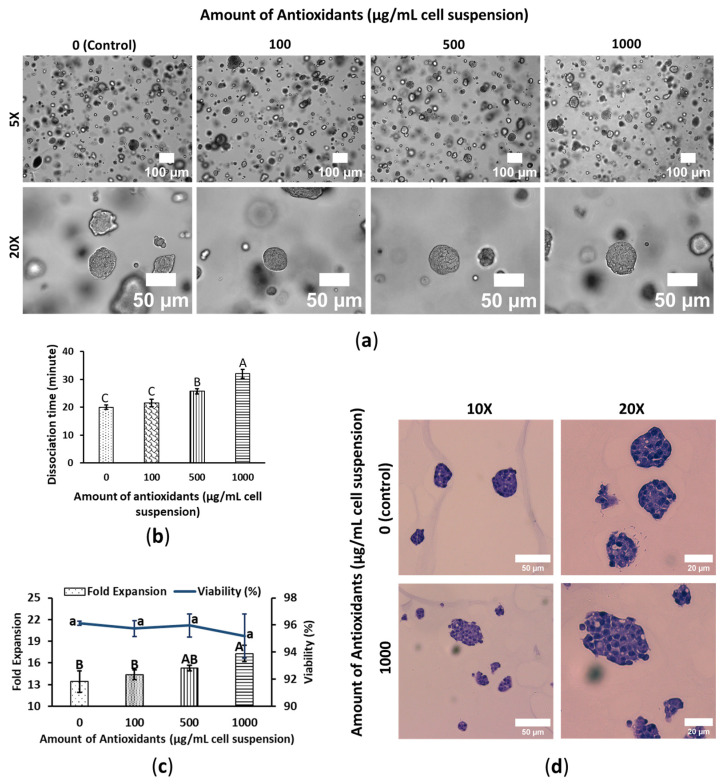
WBA elevates HepG2 liver cancer cell’s growth performance. Cells were cultured in 3D-embedded conditions using PGS (0.5%) in 24-well plates. Antioxidants were induced on day 3 and the cell was harvested on day 7. The cell spheroids shown in the figure are also on day 7. (**a**) Cell morphology of HepG2. The scale bars are 100 and 50 μm; the resolutions are 5× and 20×. (**b**) Cells dissociation time with trypsin EDTA 0.25% at 37 °C. Data are shown as means ± SD of three independent biological replicates (*n* = 3). *p* < 0.05. The means that do not share a letter are significantly different. (**c**) Fold expansion and viability of HepG2. The column chart shows fold expansion, and the line chart shows viability. Data are shown as means ± SD of three independent biological replicates (*n* = 3). *p* < 0.05. The means that do not share a letter are significantly different. The uppercase letters represent the significant difference in fold expansion and the lowercase letters represent the significant difference in viability. (**d**) Cell spheroid images of H&E stained HepG2. The scale bars are 50 and 20 μm; the resolutions are 10× and 20×.

Like WS480, HepG2 cells formed spheroids with varying sizes observed within the same treatment group. However, the cells within the spheroids were tightly packed, making individual cells indistinguishable, and there was no specific arrangement of cells. WBA treatment did not significantly alter HepG2 cell morphology; however, an increase in spheroid size was observed in a subset of spheroids at higher WBA concentrations (Figure 2a). On day 7, the spheroids were harvested and then treated with trypsin-EDTA 0.25% to dissociate them into single cells. WBA was found to influence dissociation time in HepG2 cells, with increased dissociation time correlating with higher WBA concentrations; for instance, control and 1000 μg/mL HepG2 cells required 20 ± 0.82 and 32 ± 1.64 min, respectively, for dissociation. However, lower WBA concentrations, such as 100 μg/mL, did not affect dissociation time (Figure 2b).

Cell fold expansion and viability were assessed after a 4-day exposure to WBA. Cell viability remained consistently around 95% across all treatments, with no significant differences between control and WBA-treated cells. In contrast, cells treated with 1000 µg of WBA showed significantly higher fold expansion compared to the control, with growth increasing in line with WBA concentration. Control cells had a growth rate of 13.42 ± 1.15, while cells treated with 1000 µg WBA exhibited approximately 30% higher growth, suggesting that WBA positively affects HepG2 liver cancer cells (Figure 2c). These findings indicate that WBA promotes HepG2 cell growth without exhibiting toxicity, suggesting potential antioxidant-driven, growth-enhancing properties.

Additionally, H&E staining was performed on HepG2 spheroids to examine cellular morphology, structural organization, and spatial arrangement. Control and WBA-treated HepG2 spheroids were irregular in shape and cells were evenly distributed without forming any pattern. There was no significant difference between the control and treated cells; both cells looked well hydrated and had a high nuclear-to-cytoplasm ratio (Figure 2d).

### 3.3. WBA Does Not Interfere with HeLa’s Growth Performance

**Figure 3:**
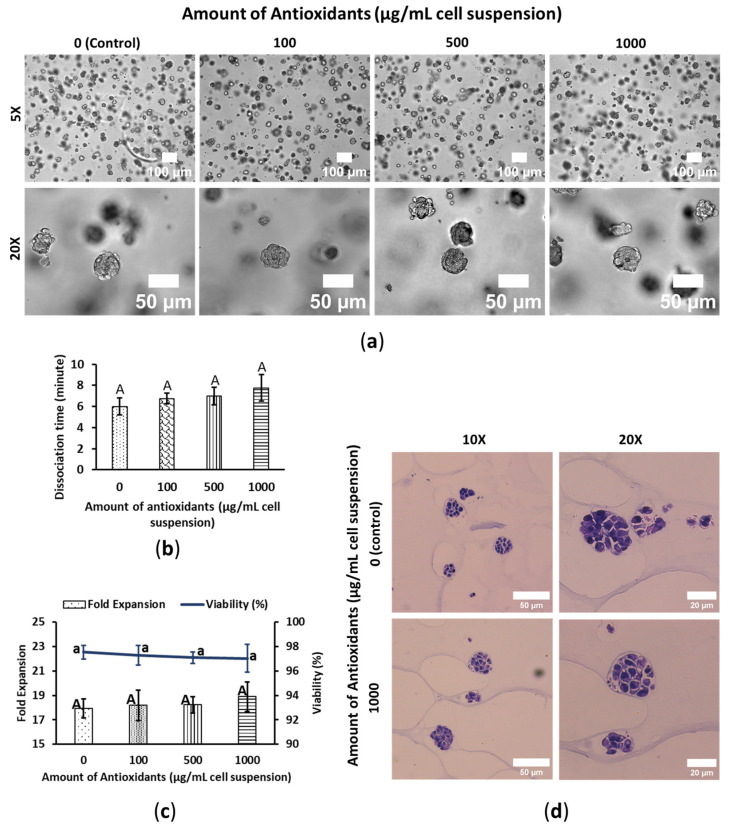
WBA does not impact HeLa cervical cancer cell’s growth performance. Cells were cultured in 3D-embedded conditions using PGS (0.5%) in 24-well plates. Antioxidants were induced on day 4 and the cell was harvested on day 8. The cell spheroids shown in the figure are also on day 8. (**a**) Cell morphology of HeLa. The scale bars are 100 and 50 μm; the resolutions are 5× and 20×. (**b**) Cell dissociation time with trypsin EDTA 0.25% at 37 °C. Data are shown as means ± SD of three independent biological replicates (*n* = 3). *p* < 0.05. The means that do not share a letter are significantly different. (**c**) Fold expansion and viability of HeLa. The column chart shows fold expansion, and the line chart shows viability. Data are shown as means ± SD of three independent biological replicates (*n* = 3). *p* < 0.05. The means that do not share a letter are significantly different. The uppercase letters represent the significant difference in fold expansion and the lowercase letters represent the significant difference in viability. (**d**) Cell spheroid images of H&E stained HeLa. The scale bars are 50 and 20 μm; the resolutions are 10× and 20×.

Hela cells formed spheroids in the 3D PGS embedded condition, though some exhibited slight deformations. Cells within the spheroids were densely packed, making individual cells indistinguishable, with no specific cellular arrangement. WBA treatment did not significantly affect the morphology of the HeLa spheroids, and no cell precipitation was observed in either treated or untreated groups (Figure 3a). During harvesting, dissociation times were also recorded to evaluate the impact of WBA on cell dissociation time. WBA had no noticeable effects on HeLa cell spheroid dissociation times; control and 1000 μg/mL HeLa cells had dissociation times of 6 ± 0.81 and 7.75 ± 1.25 min, respectively (Figure 3b).

Cell fold expansion and viability were assessed after a 4-day exposure to WBA. Viability remained consistently around 97% across all treatments, with no significant differences between control and WBA-treated cells. Although there was a slight increase in fold expansion in the WBA-treated group, this increase was not statistically significant compared to the control (Figure 3c). HeLa may activate cellular pathways (e.g., Nrf2 signaling) that increase the expression of detoxifying enzymes and protective molecules [28]. This suggests that WBA does not affect the growth of cervical cancer HeLa cells and does not exhibit any toxic effects on them.

HeLa cells were also stained with H&E to examine cell spheroid anatomy. Cells adhered to one another with small gaps between them in the spheroids, with no distinct arrangement. Both control and WBA-treated cells displayed hyperchromatic nuclei and potentially higher nuclear-to-cytoplasmic ratios. Therefore, there were no significant differences in cellular morphology between WBA-treated and untreated cells (Figure 3d).

## 4. Discussion

WBA extract was tested on three human cancer cell lines to assess its cytotoxic effects. In SW480 cells, the observed morphological alterations, such as cell shrinkage and membrane blebbing, are likely linked to WBA-induced oxidative stress and disruption of intracellular water balance [13]. Previous studies have demonstrated that natural antioxidants, such as those derived from wheat bran, sorghum bran, coffee grounds, and rice bran, can generate ROS, leading to oxidative stress in cancer cells [29,30,31]. This stress triggers morphological changes, including cellular dehydration, possibly due to damage to the cell membrane or cytoskeletal components, impairing the ability to regulate osmotic pressure [14,32]. Increased dissociation times in WBA-treated cells may reflect reduced structural integrity and cytoskeletal reorganization. The reduced nuclear–cytoplasmic ratio and condensed cytoskeleton observed in these cells further suggest apoptosis pathway activation [33]. WBA’s ability to suppress fold expansion aligns with findings from other polyphenolic antioxidants, which have been shown to disrupt cell cycle progression and induce apoptosis in cancer cells [29,30,31,32]. ROS generated by antioxidants can damage DNA, impair mitochondrial function, and activate pro-apoptotic signaling, contributing to cell death [9,11]. WBA may modulate the Wnt/β-catenin signaling pathway, which is often aberrantly activated in colorectal cancer. Phenolic compounds present in WBA, such as ferulic acid, can influence this pathway by promoting the degradation of β-catenin, thereby reducing its nuclear translocation and subsequent transcriptional activation of oncogenes like *c-Myc* and *Cyclin D1* [34], resulting in the inhibition of cell proliferation. These findings suggest WBA holds potential as a therapeutic agent for colon cancer, warranting further exploration of its molecular mechanisms and efficacy in in vivo models.

In contrast, WBA promoted growth in HepG2 cells, highlighting the variability in antioxidant responses across cancer types. This phenomenon may stem from the liver’s natural exposure to antioxidants and its robust detoxification mechanisms, which make HepG2 cells more adaptable to antioxidant-induced stress [35,36,37]. Liver enzymes like cytochrome P450 can oxidize toxins, increasing their reactivity, where molecules such as glutathione attach to these reactive intermediates, rendering them water-soluble for excretion [38]. Additionally, WBA’s interaction with adhesion molecules or the extracellular matrix could account for the observed increase in spheroid dissociation time [39]. Similar findings in other cancer models suggest that antioxidants can modulate adhesion molecule expression, altering cellular dynamics [40,41]. The differential response between HepG2 and SW480 cells underscores the importance of cell-type specificity in antioxidant-based treatments. While WBA promotes growth in liver cancer cells, its ability to modulate oxidative stress and cellular behavior highlights its potential for therapeutic applications, provided its effects are carefully managed in specific cancer contexts.

In HeLa spheroids, WBA exhibited neither cytotoxicity nor significant morphological changes during the 4-day exposure. This lack of effect may result from cervical cancer cells’ enhanced tolerance to oxidative stress, supported by high endogenous antioxidant enzyme levels, such as superoxide dismutase and catalase [42,43]. Additionally, concentration and exposure duration may have been insufficient to elicit cytotoxicity, suggesting that HeLa cells may require higher doses or longer exposure to reveal any potential effects. The structural complexity of the 3D spheroid culture model likely contributes to the observed resistance, as the cell–cell and cell–matrix interactions provide a more protective environment against oxidative challenges [44,45].

The unique responses of these cancer cell lines—cytotoxicity in SW480, growth promotion in HepG2, and no significant impact on HeLa—emphasize the complexity of antioxidant interactions with cancer cells. Factors such as cellular redox state, antioxidant defense mechanisms, and tissue origin must be considered when evaluating potential therapeutic applications. WBA’s selective effects highlight its promise for targeted cancer therapies, although further research is necessary to elucidate its mechanisms and optimize its therapeutic potential. The findings from the study might be limited by batch variability of wheat bran sources such as wheat variety, cropping location, or extraction methods. The discovery from this research warrants future in vivo studies with animal models to further identify application doses, time courses, and therapeutic strategies.

## 5. Conclusions

This study provides comprehensive insights into the effects of WBA on SW480, HepG2, and HeLa cancer cell lines within a 3D-embedded PGS matrix system. WBA exhibited selective effects, inhibiting growth in SW480 colon cancer cells through oxidative stress-induced apoptosis, promoting growth in HepG2 liver cancer cells, likely due to their robust detoxification mechanisms, and having no significant impact on HeLa cervical cancer cells. These findings highlight the complexity of antioxidant interactions with cancer cells and underscore WBA’s potential for targeted, personalized cancer therapies. Further research is necessary to elucidate the underlying molecular mechanisms, optimize dosing strategies, and evaluate its efficacy in in vivo models. Additionally, exploring WBA’s synergy with conventional chemotherapies could provide valuable insights into combination treatments that enhance cancer therapy outcomes. This approach may offer synergistic effects tailored to specific cancers, such as augmenting chemotherapy efficiency in colon cancer or mitigating toxicity in liver cancer, thereby advancing personalized and more effective treatment strategies.

## Figures and Tables

**Table 1:** WBA extract concentration, the volume of WBA extract induction, and the volume of fresh medium addition in the 3D-embedded condition in a 24-well plate (24-WP).

SN	WBA Extract Concentration (µg/mL Cell Suspension) ^1^	In 24-WP	2% WBA Extract and Fresh Medium Addition
01	100	200 µg/2 mL/well	10 uL 2% WBA extract with 1500 uL fresh medium
02	500	1000 µg/2 mL/well	50 uL 2% WBA extract with 1500 uL fresh medium
03	1000	2000 µg/2 mL/well	100 uL 2% WBA extract with 1500 uL fresh medium

^1^ To select the concentration of WBA extract, we aimed to encompass a range from low to high doses to observe potential dose-dependent effects. Previous studies have employed varying concentrations of wheat bran extracts to evaluate their anticancer properties [25,26].

## Data Availability

The original contributions presented in the study are included in the article. Further inquiries can be directed to the corresponding author.
